# Sexual Experiences and Sexuality of Forensic Mental Health Patients - A Theoretical Framework

**DOI:** 10.1192/j.eurpsy.2024.348

**Published:** 2024-08-27

**Authors:** V. Šendula Jengić, P. Cargonja

**Affiliations:** ^1^Forensic Psychiatry; ^2^County hospital Insula, Rab, Croatia

## Abstract

**Introduction:**

Sexuality and sexual experiences in forensic mental health patients are intricate issues at the crossroads of mental health, legal contexts, and societal perceptions. Forensic mental health patients, situated within the criminal justice system, require psychiatric treatment due to offenses committed. Understanding their sexual behaviors, experiences, and attitudes towards sexuality is pivotal for effective therapeutic interventions and rehabilitation. Various factors influence the sexuality of forensic mental health patients. Mental health disorders impact an individual’s sexual expression, and medications used to treat these conditions may affect libido, sexual functioning, and arousal patterns, posing challenges in their sexual experiences.

**Objectives:**

Addressing the sexual needs and experiences of these individuals requires a comprehensive approach. Mental health professionals must create safe and non-judgmental spaces for patients to openly discuss their sexual concerns. Therapeutic interventions should focus on facilitating healthy sexual expression and providing education on consent, healthy relationships, and understanding boundaries. Forensic mental health professionals often receive specialized training to address the complex intersection of sexuality and mental health within legal contexts. Navigating ethical and legal boundaries while providing support to these individuals is crucial, ensuring that interventions align with legal regulations and ethical standards. Rehabilitation programs in forensic mental health facilities should integrate sex education and relationship-building skills to help patients develop a healthy understanding of sexuality. These programs aim to reduce reoffending and support the reintegration of individuals into society by promoting responsible and respectful sexual behaviors.

**Methods:**

This study conducts a systematic literature review to comprehend the intricate nature of sexuality and sexual experiences among forensic mental health patients.

**Results:**

Research in this area faces limitations and ethical challenges due to the sensitivity of the topic. Ethical considerations, such as confidentiality and consent, must be meticulously addressed in studies and while providing care to this population.

**Conclusions:**

Understanding the sexuality and sexual experiences of forensic mental health patients is integral to their treatment and rehabilitation. It requires a multifaceted approach that acknowledges the complexities these individuals face due to mental health conditions, personal histories, and the nature of their care environment. Tailored and comprehensive support can promote healthier sexual behaviors, relationships, and overall well-being among this population.

**Disclosure of Interest:**

None Declared

